# From proteome to candidate vaccines: target discovery and molecular dynamics-guided multi-epitope vaccine engineering against kissing bug

**DOI:** 10.3389/fimmu.2024.1413893

**Published:** 2024-06-10

**Authors:** Faisal F. Albaqami, Ali Altharawi, Hassan N. Althurwi, Khalid M. Alharthy

**Affiliations:** ^1^ Department of Pharmacology and Toxicology, College of Pharmacy, Prince Sattam Bin Abdulaziz University, Al-Kharj, Saudi Arabia; ^2^ Department of Pharmaceutical Chemistry, College of Pharmacy, Prince Sattam Bin Abdulaziz University, Al-Kharj, Saudi Arabia

**Keywords:** immunoinformatics, candidate vaccine, *Trypanosoma cruzi*, molecular modeling, MD simulations

## Abstract

**Introduction:**

*Trypanosoma cruzi* is a protozoan parasite that causes the tropical ailment known as Chagas disease, which has its origins in South America. Globally, it has a major impact on health and is transported by insect vector that serves as a parasite. Given the scarcity of vaccines and the limited treatment choices, we conducted a comprehensive investigation of core proteomics to explore a potential reverse vaccine candidate with high antigenicity.

**Methods:**

To identify the immunodominant epitopes, T. cruzi core proteomics was initially explored. Consequently, the vaccine sequence was engineered to possess characteristics of non-allergenicity, antigenicity, immunogenicity, and enhanced solubility. After modeling the tertiary structure of the human TLR4 receptor, the binding affinities were assessed employing molecular docking and molecular dynamics simulations (MDS).

**Results:**

Docking of the final vaccine design with TLR4 receptors revealed substantial hydrogen bond interactions. A server-based methodology for immunological simulation was developed to forecast the effectiveness against antibodies (IgM + IgG) and interferons (IFN-g). The MDS analysis revealed notable levels of structural compactness and binding stability with average RMSD of 5.03 Aring;, beta-factor 1.09e+5 Å, Rg is 44.7 Aring; and RMSF of 49.50 Aring;. This is followed by binding free energies calculation. The system stability was compromised by the complexes, as evidenced by their corresponding Gibbs free energies of -54.6 kcal/mol.

**Discussion:**

Subtractive proteomics approach was applied to determine the antigenic regions of the T cruzi. Our study utilized computational techniques to identify B- and T-cell epitopes in the T. cruzi core proteome. In current study the developed vaccine candidate exhibits immunodominant features. Our findings suggest that formulating a vaccine targeting the causative agent of Chagas disease should be the initial step in its development.

## 1 Introduction

There are just a few hematophagous subfamilies of Reduviidae, called Triatominae, or kissing bugs (1). *Trypanosoma cruzi* is the parasite that they carry, which allows them to spread Chagas disease. With an estimated six to seven million cases globally and 12,000 fatalities every year (2), Chagas disease is a potentially fatal condition. Taxonomic studies on vectors may be one facet of the long-running efforts to curb the disease’s spread as a public health issue.

Propagation of *T. cruzi* and related mortality have historically been centered in rural, difficult-to-live-in areas of Latin America that host infection vectors. About 20 million individuals are afflicted with this parasitic illness, which is mostly found in the tropical regions of Central and South America. Furthermore, the majority of those affected in Central America are immigrants from endemic parts of Latin America (3). *T. cruzi*, the parasite that causes Chagas disease, is thought to infect 6–7 million individuals globally (4). Infected people have been sent to Canada and Europe in recent decades, as well as other locations inside and outside of Latin America (5–7). This made the illness more widely acknowledged as a public health issue on a global scale and made it easier to combat false information, a lack of societal demand, and a lackluster governmental commitment to finding solutions for Chagas disease-related issues (8).

At specific times of its developmental cycle, the kinetoplastid protozoan infecting vertebrates as well and invertebrates (5). Once a triatomine vector has fed on blood from a mammalian host that is carrying the parasite, it consumes the circulating trypomastigotes (7). The significant stage of reproduction in the invertebrate hosts, epimastigotes, are produced by trypomastigotes, which are present in the midgut of the vector (9). Once in the hindgut, epimastigotes change into pathogenic metacyclic trypomastigotes. The vector’s excrement then contains these metacyclic trypomastigotes that are released from its body. Through bite wounds or intact mucosal membranes, metacyclic trypomastigotes penetrate their mammalian host and use lysosomes to infiltrate different nucleated cells (10). Intracellular amastigotes develop from trypomastigotes in the cytoplasm, which have a doubling duration of around 12 hours and proliferate for 4–5 days. Subsequently, the amastigotes transform into trypomastigotes, which are then discharged into the circulation, and the host cell breaks. The migratory pathogens may now infiltrate cells, infect prey vectors, and start fresh replication cycles. If effective anti-trypanosomal medication is not administered, the mammalian host remains infected for the duration of its life (3).

Tc52, a virulence factor that is essential to the infection process, is one of the proteins released by *T. cruzi.* Using a peptide sequence, researchers performed molecular profile analysis of the Tc52 minimum sequence that has an immunosuppressive effect. They then integrated *in vitro* and *in vivo* approaches to demonstrate the sequence’s function in the progression of infection. The likelihood of developing vaccines or drugs to combat *T. cruzi* has increased with the identification of this factor’s biological function (11, 12).

Benznidazole and nifurtimox are the two most often prescribed medications for this illness, and their suggested course of therapy varies from 60 to 90 days (13). Extended therapy sessions may be financially and logistically burdensome for impoverished individuals with limited access to medical care. Due to the potentially fatal side effects of these drugs, therapy must be stopped often (14–16). The effectiveness of a prophylactic DNA vaccine incorporating T. cruzi genes was evaluated in a prior study using dogs with Chagas disease that was created artificially (17).

Recently, there has been an increasing interest in combining immunoinformatics with the subtractive proteomics technique to develop a cost-effective and effective vaccination against different infections. In this study, a reverse vaccinology-based immunoinformatics pipeline was employed to screen possible virulent/antigenic proteins for the identification of epitopes from the complete proteome of *T. cruzi*, utilizing subtractive proteomics. In contrast to conventionally developed vaccines, immunoinformatics vaccines exhibit thermodynamic stability and are associated with reduced or negligible side effects (18). Therefore, we have made significant advancements in the development of a highly effective MEV targeting S. aureus. We have conducted various bioinformatics techniques, including protein-protein docking, MMGBSA binding free energy analysis, molecular dynamics (MD) simulations, simulation of the vaccine-induced immune response, and computational expression validation through in silico cloning, to confirm its stability and effectiveness. The present work revealed that the proposed MEV can establish stable contacts with human immune receptors and effectively stimulating a robust immune system response in the host.

## 2 Methodology

**Figure 1:**
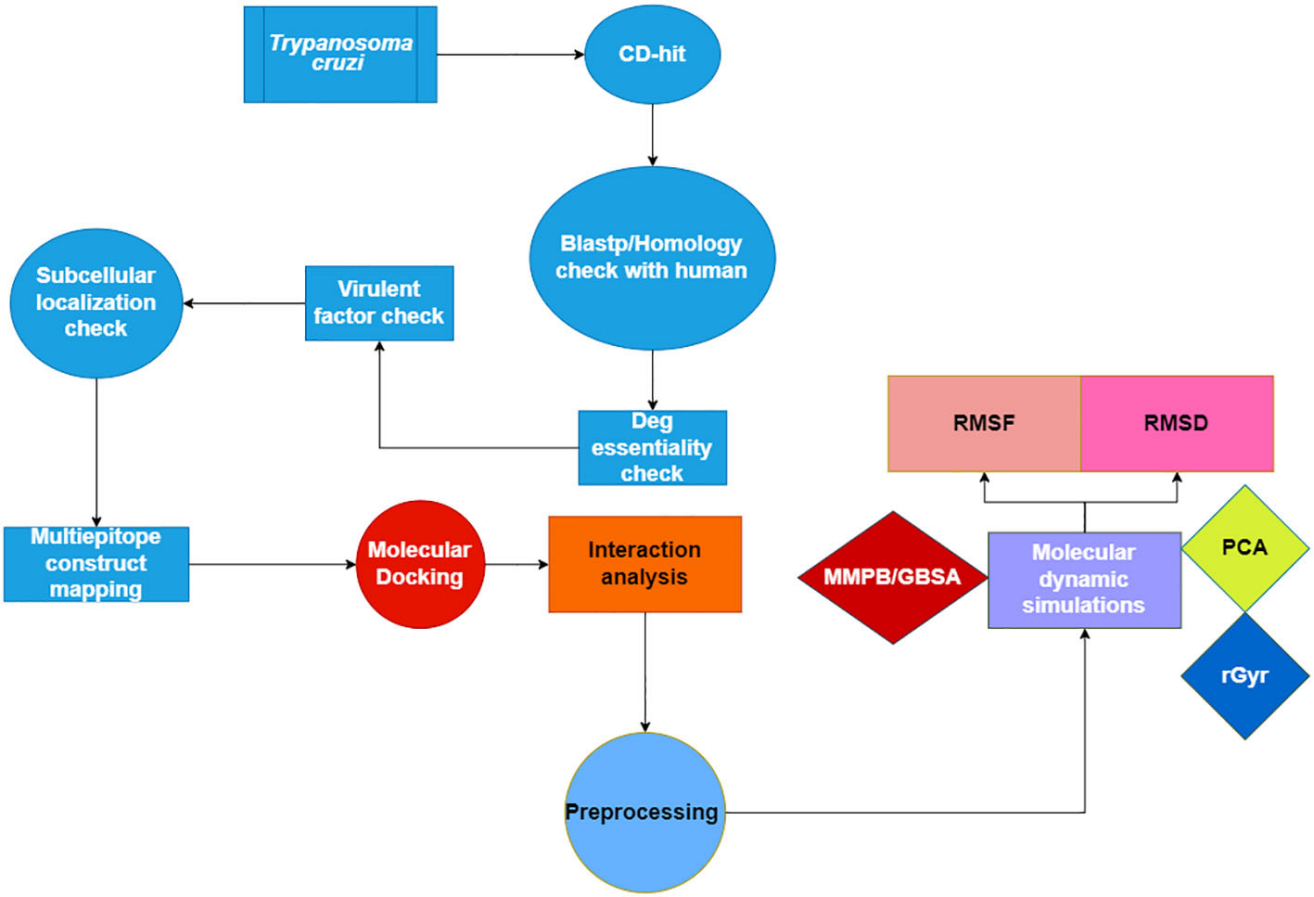
Flow chart of the current study presenting the multi-epitope vaccine construct using Insilico approach.

**Figure 1** displays a conceptual scheme of the study’s approach.

### 2.1 The approach of subtractive proteomics

Subtractive proteome analysis is used to the core proteome to identify potential new targets for immunization https://www.ncbi.nlm.nih.gov/datasets/taxonomy/5693/. The removal of redundant sequences is the first step in subtractive proteomics. Nucleotide or protein sequences are compared and arranged in the Cluster Database at High Identity with Tolerance (CD-HIT) to get rid of unnecessary information and boost sequence analysis performance. The most often used program is CD-HIT, which reduces sequence mismatches. In this experiment, we filtered the whole core proteome at 80% confidence level using the CD-HIT server. It aims to lessen repetition by following a user-defined sequence identity threshold (19). Proteins obtained from the CD-HIT service were used in a BlastP search to identify a non-redundant database which includes non-homologous T. cruzi proteins. By matching the query protein with the database that the user has selected, Protein-Protein BLAST, also known as BlastP, finds the sequences of proteins in the other database which most closely resemble the query (20). Proteins were considered non-homologous if their identity was more than 30% and their query coverage was greater than 70%. Understanding how a certain protein functions is necessary to develop vaccinations that are effective. One method that may assist in figuring out a protein’s biological function is to forecast where it will be located inside the cell. Additionally, studies have shown that localization is important in developing vaccine candidates since proteins are contained in several locations. The psortb platform was used to forecast the subcellular localization of non-homologous proteins (21). Because of their important part in the pathophysiology of the illness, virulence proteins are essential (22). The label “virulent” was used to *T. cruzi* homologs with bit scores higher than 100 and identities higher than 30%. A TMHMM server was used to identify transmembrane helices. The presence or absence of transmembrane helices in a protein may be ascertained using a program known as TMHMM (Transmembrane Helices; Hidden Markov Model) (23). When developing a vaccine, we gave priority to the most antigenic proteins devoid of transmembrane helices. Afterwards, the antigenicity of pathogenic proteins was evaluated using the Vaxijen server (24). The antigenic proteins that scored highest on antigenicity were selected as potential candidates for vaccination. Moreover, the Protparam tool was utilized to determine the molecular properties of the proteins, and the AllerTOP software was utilized to evaluate the allergenicity of the proteins (25, 26).

### 2.2 Prediction and assessment of epitopes

#### 2.2.1 Evaluating the antigenic properties

There are two primary proteins that make up the *Trypanosoma cruzi* pathogenic: the mucin-associated surface protein 1 (MASP) and MASP 2. The previously described *Trypanosoma cruzi* bacterial proteins were evaluated for antigenicity and allergenicity utilizing online tools, namely VaxiJen (http://www.ddg-pharmfac.net/vaxijen/) (24) for antigenicity and AllerTOP (https://www.ddg-pharmfac.net/AllerTOP/) (25) for allergenicity, in order to progress the processing of the proteins for the creation of a Multi-Epitope Subunit Vaccine (MESV). To creating a multi-epitope subunit vaccine, the (MASP) protein were chosen due to their high antigenicity and non-allergenicity. An immunoinformatic process was used to find appropriate epitopes for the creation of a multi-epitope vaccine. The protein sequences of the mucin-associated surface protein (MASP) family protein were retrieved from the publicly accessible NCBI database (27). To find proteins with high immunogenicity and epitopes for the vaccine’s development, a series of separate steps in the analytical process are depicted in **Figure 1**.

#### 2.2.2 Protein epitope screening

An analysis of the mucin-associated surface protein (MASP), [*Trypanosoma cruzi* strain CL Brener] was carried out using an online program called NetCTL1.2 (https://services.healthtech.dtu.dk/services/NetCTL-1.2) (28), to identify putative immunological targets like epitopes capable of stimulating cytotoxic T lymphocytes and eliciting a strong immune response. The major histocompatibility complex (MHC I) is one of the key components that the specified server uses to forecast binding, proteasomal C-terminus cleavage, and transport efficiency. By using a default threshold of 0.75, these parameters help identify highly immunogenic and effective epitopes. After probable CTL epitopes were identified, helper T lymphocyte activation potential was predicted using the IEDB MHC-II module (http://tools.iedb.org/mhcii/) (29). Seven different human HLAs were used in this assessment. Percentile rank is used by the IEDB MHC-II module to forecast the binding affinity of HTL epitopes with MHC-II. While a lower binding affinity is correlated with a higher percentile rank, a higher percentile rank relates to a lower binding affinity. Furthermore, to predict B cell epitopes, the web-based application ABCPred (http://ailab.cs.iastate.edu/abcpreds/) was used. This website predicts the antigenicity of B cell epitopes using an SVM model and an amino acid pairs (AAP) approach (30). By taking the VaxiJen server’s expected antigenicity into account, we were able to reduce the number of B cell, HTL, and CTL epitopes that we chose. The highly antigenic epitopes were then further optimized to produce a vaccine with increased immunogenicity.

#### 2.2.3 Engineering and validation of multi-epitope vaccines

To create the most immunogenic MESV, immunogenic linkers were used to connect the desired epitopes from the mucin-associated surface protein (MASP). The CTL epitopes were connected using AAY linkers, whereas the B cell and HTL epitopes were connected using KK and GPGPG linkers, respectively (31). The linkers are immunogenic and function to prevent the expected epitopes from folding. With the help of the EAAK linker, Cholera toxin B subunit was added as an adjuvant at the N-terminal end of the vaccine construct to improve their immunogenic potential (32, 33). Next, the VaxiJen online tool (http://www.ddg-pharmfac.net/vaxijen/VaxiJen/VaxiJen.html) (24) and the AllerTop tool (https://www.ddg-pharmfac.net/AllerTOP/) (25) were used to evaluate the immunogenicity and antigenicity of the developed vaccines. Additionally, the EXPASY ProtParam tools (https://web.expasy.org/protparam/) were utilized to analyze the physical and chemical properties of the designed vaccines. This online resource can predict various parameters such as molecular size, lifespan, theoretical isoelectric point (PI), GRAVY, and aliphatic index (26).

#### 2.2.4 Testing and designing the modified vaccine structure

For modelling reasons, the 3D structures of the developed vaccines (mucin-associated surface protein (MASP), were uploaded to the publicly accessible site Robetta server (https://robetta.bakerlab.org/) (34). This server has been recognized as one of the most reliable resources for tertiary structure prediction since 2014. Continued automatic model evaluation (CAMEO) is used to accomplish. Moreover, Inter-atomic distances are evaluated by this server, which gives a score of 100 for favorable evaluations and 0 for unfavorable evaluations. Additionally, Using the PyMOL program, the final vaccine construct modeled structure was made visible (35). The five models produced by Robetta for vaccine construct were assessed using the ProSA-web tool (https://bio.tools/prosa-web) (36) and finally, Using PROCHECK (https://servicesn.mbi.ucla.edu/PROCHECK/), vaccination models were further evaluated, and the top model was selected in accordance with the quality scores supplied by the aforementioned servers.

#### 2.2.5 Binding study of vaccine-TLR4

To generate strong immunogenic responses, human Toll-like receptor TLR4 must form a strong binding network with specially engineered vaccines (37). Thus, using the HDOCK web server (http://hdock.phys.hust.edu.cn/), the vaccine construct was docked with TLR4. Unlike other servers, the HDOCK server analyzes protein-protein, protein-DNA, and protein-RNA interactions using a template-based hybrid method (38). A model with ideal dimensions was selected from the group of the top ten models generated by the HDOCK server. The relationship between the constructed vaccine and the receptor (TLR-4) guided this choice. We uploaded the docking complex to the PDBsum web server (http://www.ebi.ac.uk/thornton-srv/databases/pdbsum/Generate.html) in order to see the bonding interface between the created vaccines and human TLR-4 (39). We used the PRODIGY web server (https://wenmr.science.uu.nl/) to calculate the dissociation constant (KD) value to confirm the strength of the bonding network inside the vaccine-TLR-4 complex (40).

#### 2.2.6 Molecular dynamic simulations

The dynamic behavior of docked complexes was observed by the utilization of molecular dynamics (MD) simulations. The AMBER16 software is utilized for doing simulations and subsequent analysis using its various modules (41). The tleap program in AMBER16 is utilized for constructing topology files and incorporating missing atoms. The solvation of the system was conducted using three-point convertible intermolecular potential (TIP3P) water. The force fields utilized for mathematical calculations were GAFF (42) and ff14SB (43). The examination of MD simulation trajectories was conducted using the AMBER PTRAJ module. The analysis consists of several metrics, including RMSD (Root Mean Square Deviation), RMSF (Root Mean Square Fluctuation), Rg (Radius of Gyration). To do graphical analysis and evaluation of certain parameters, a two-dimensional (2D) plotting tool called “Xmgrace” is employed. Hence, to facilitate efficient sampling and folding kinetics, it is imperative that the energy and kinetics of hydrogen bonding are optimized. This imparts stability to the structure of proteins and the selectivity necessary for selected interactions with macromolecules (44). The time-dependent hydrogen bond diagram was generated using the cpptraj module of AMBER16. This observation was consistent with the outcome of the PCA analysis followed by MMPB/GBSA analysis.

### 2.3 Analysis of interactions

The analysis of docked complexes is significantly enhanced by the inclusion of interaction analysis. This aids in the identification of the orientation and spatial arrangement of the vaccine construct within the binding site. Moreover, it is beneficial to establish the stability of the complex. Both hydrogen and Van der Waals interactions are incorporated in the interaction study. The phenomenon of hydrogen bonding exhibits a distinct directionality and contact specificity between a protein target and vaccine construct, which can be considered a fundamental component of molecular recognition (45, 46).

### 2.4 Binding free energies

The molecule’s binding free energy is the total of its Gsolv, Ggas, Van der Waals, and electrostatic interaction energies (47). MMPBSA/GBSA based binding free energy estimates were performed on final 100 frames that were acquired from the 100 ns of MD simulations to analyze the binding affinity. Determining the variation in free energy between the solvated and unsolvated phases was the main goal of this work (48). More specifically, the deduced energy of interactions of complex connecting residues is compared to the free energy of two distinct state conformations. To determine the free energy of our anticipated complex, ΔGbind, solv was computed using the equations shown below:


ΔG(bind) = ΔG(solv) + ΔE(MM) + ΔG(SA)

The variation in GBSA solvation energy is denoted by ΔGsolv. A distinction in the minimized energies is referred to as ΔEMM. ΔGSA represents the variation between the complex’s surface area energy and the total of all surface area energies.

## 3 Results and discussion

### 3.1 Subtraction of the proteome

**Figure 2:**
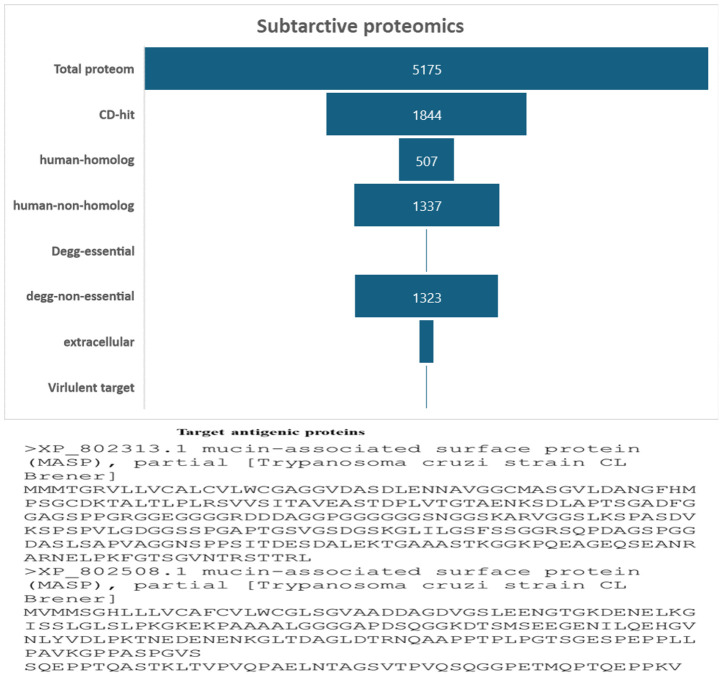
The diagram illustrates the subcellular distribution of crucial proteins inside the cellular structure. Only 1% of the proteins are reported to be in extracellular space.

In this work, a subtractive proteomics methodology was used to forecast potential candidates for the creation of MEVs targeting *Trypanosoma cruzi*. The process gradually removes proteins that are deemed less acceptable from the whole of the *Trypanosoma cruzi* proteome. A proteome of 5175 proteins from the *Trypanosoma cruzi* “ASM20906v1” was obtained from https://www.ncbi.nlm.nih.gov/datasets/taxonomy/5693/. The proteome underwent CD-HIT filtering with a threshold of 80% (0.8). Forty-nine (3331) paralogous proteins were excluded by the CD-HIT suite, leaving 1844 non-paralogous proteins for further investigation **Figure 2**. Subsequently, electronic graphs (EGs) were examined to identify non-paralogous proteins using a threshold of 10−5 e-value. The study revealed a total of 9 essential proteins, which were further examined for their similarity with the human proteome (Taxonomic id: 9606). To avoid an autoimmune reaction that might target and damage its own cells by recognizing them as foreign particles, it is crucial to remove human homologs. To mitigate such a scenario, it is essential to refrain from using homologous proteins. A grand total of 502 human homologs were identified and subsequently eliminated, while further examinations were conducted on the 1337 non-homologous proteins. The Psortb server was used to estimate the cellular distribution of important proteins. The analysis revealed that 130 proteins were anticipated to be in the extracellular region, (see **Figure 2**). The proteins located in the cytoplasm were removed, and the proteins that remained were chosen for further analysis.

### 3.2 Identification of T-cell epitopes within selected B-cell epitopes

The web-based program ABCPred was applied to predict B cell epitopes as shown in **Table 1**. The binding of peptides to major histocompatibility complexes (MHC) plays a crucial role in determining the unique cellular immunogenicity within the framework of T-cell-mediated immune responses. The present investigation reveals the identification of eight T-cell epitopes that exhibit interactions with both major histocompatibility complex (MHC)-I and MHC-II types. Cytotoxic T lymphocytes (CTL) are key components of the cellular immune response, since they possess the ability to identify immunogenic antigens present on the surfaces of cells that have been infected by viruses. The antigen-specific T-cell receptor exhibits binding affinity towards CTL epitopes and MHC class-I molecules, resulting in the formation of complexes on the surfaces of cells infected with the virus. As a result, our prediction yielded 9-mer CTL epitopes, and we identified four possible CTL epitopes that specifically target the human allele HLA-A*68 to01.

**Table 1:** Epitopes shared between IEDB and BCPreds web server predictions, specifically selected for B cells.

Rank	Sequence	Start position	Score
1	DVKSPSPVLGDGGSSP	146	0.93
2	GGEGGGGRDDDAGGPG	107	0.92
3	CVLWCGAGGVDASDLE	14	0.9
4	PVLGDGGSSPGAPTGS	152	0.89
4	GSPPGRGGEGGGGRDD	101	0.89
5	AGEQSEANRARNELPK	237	0.88
5	KGLILGSFSSGGRSQP	174	0.88
5	GGPGGGGGGSNGGSKA	119	0.88
6	GRSQPDAGSPGGDASL	185	0.86
7	SGVLDANGFHMPSGCD	39	0.85
8	GSSPGAPTGSVGSDGS	158	0.83
9	SNGGSKARVGGSLKSP	128	0.82
10	DPLVTGTAENKSDLAP	76	0.81
11	NSPPSITDESDALEKT	208	0.8
12	GDASLSAPVAGGNSPP	196	0.79
13	TAENKSDLAPTSGADF	82	0.78
13	GFHMPSGCDKTALTLP	46	0.78
13	TGSVGSDGSKGLILGS	165	0.78
14	GGSLKSPASDVKSPSP	137	0.75
15	VGGCMASGVLDANGFH	33	0.74
15	NELPKFGTSGVNTRST	248	0.74
15	AGGVDASDLENNAVGG	20	0.74
16	LAPTSGADFGGAGSPP	89	0.7
17	TDESDALEKTGAAAST	214	0.65
18	LRSVVSITAVEASTDP	62	0.64
19	DKTALTLPLRSVVSIT	54	0.57
1	LGGGGAPDSQGGKDTS	69	0.94
2	VQSQGGPETMQPTQEP	190	0.92
3	SGESPEPPLLPAVKGP	138	0.88
4	GKDTSMSEEGENILQE	80	0.87
4	AFCVLWCGLSGVAADD	13	0.87
4	KGLTDAGLDTRNQAAP	115	0.87
4	EDENENKGLTDAGLDT	109	0.87
5	GVSSQEPPTQASTKLT	158	0.85
6	ASTKLTVPVQPAELNT	168	0.84
6	QAAPPTPLPGTSGESP	127	0.84
7	AGDVGSLEENGTGKDE	29	0.83
8	LEENGTGKDENELKGI	35	0.81
8	GVAADDAGDVGSLEEN	23	0.81
9	PAVKGPPASPGVSSQE	148	0.79
10	NLYVDLPKTNEDENEN	99	0.77
11	ENILQEHGVNLYVDLP	90	0.76
12	AGSVTPVQSQGGPETM	184	0.72
13	GKEKPAAAALGGGGAP	60	0.69
14	GISSLGLSLPKGKEKP	49	0.61
15	VMMSGHLLLVCAFCVL	2	0.57
16	KDENELKGISSLGLSL	42	0.53

Helper T lymphocytes (HTL) play a pivotal role in the adaptive immune response by eliciting antibody secretion from B-cells and facilitating the activation of alternative T-cells. This study employed the NN-align algorithm from the IEDB server to predict four HTL epitopes that specifically target the human allele HLA-DRB1*15:01. The antigenicity, allergenicity, and toxicity of the anticipated epitopes were assessed and are presented in **Table 2**.

**Table 2:** Epitopes chosen for both MHC molecules, targeting both B and T cells.

MASP1 CTL	Antigenicity	Toxicity	Allergenicity	Score
ASDLENNAV	Antigen	Non-toxic	Non-allergen	0.9588
GSDGSKGLI	Antigen	Non-toxic	Non-allergen	1.2036
MMTGRVLLV	Antigen	Non-toxic	Non-allergen	0.6158
MASP1 HTL				
MTGRVLLVCALCVLW	Antigen	Non-toxic	Non-allergen	0.5148
TALTLPLRSVVSITA	Antigen	Non-toxic	Non-allergen	0.6364
GRVLLVCALCVLWCG	Antigen	Non-toxic	Non-allergen	0.9023
B cell epitopes				
PVLGDGGSSPGAPTGS	Antigen	Non-toxic	Non-allergen	1.2261
AGEQSEANRARNELPK	Antigen	Non-toxic	Non-allergen	0.6393
KGLILGSFSSGGRSQP	Antigen	Non-toxic	Non-allergen	1.5337
MASP2 CTL				
MMSGHLLLV	Antigen	Non-toxic	Non-allergen	0.8079
VMMSGHLLL	Antigen	Non-toxic	Non-allergen	1.2625
SLEENGTGK	Antigen	Non-toxic	Non-allergen	1.0472
MASP2 HTL				
GKDENELKGISSLGL	Antigen	Non-toxic	Non-allergen	0.638
GHLLLVCAFCVLWCG	Antigen	Non-toxic	Non-allergen	1.0684
NTAGSVTPVQSQGGP	Antigen	Non-toxic	Non-allergen	0.5518
B cell epitopes				
SGESPEPPLLPAVKGP	Antigen	Non-toxic	Non-allergen	0.63
GVSSQEPPTQASTKLT	Antigen	Non-toxic	Non-allergen	0.8337
ASTKLTVPVQPAELNT	Antigen	Non-toxic	Non-allergen	0.6613

### 3.3 Vaccine construction

**Figure 3:**
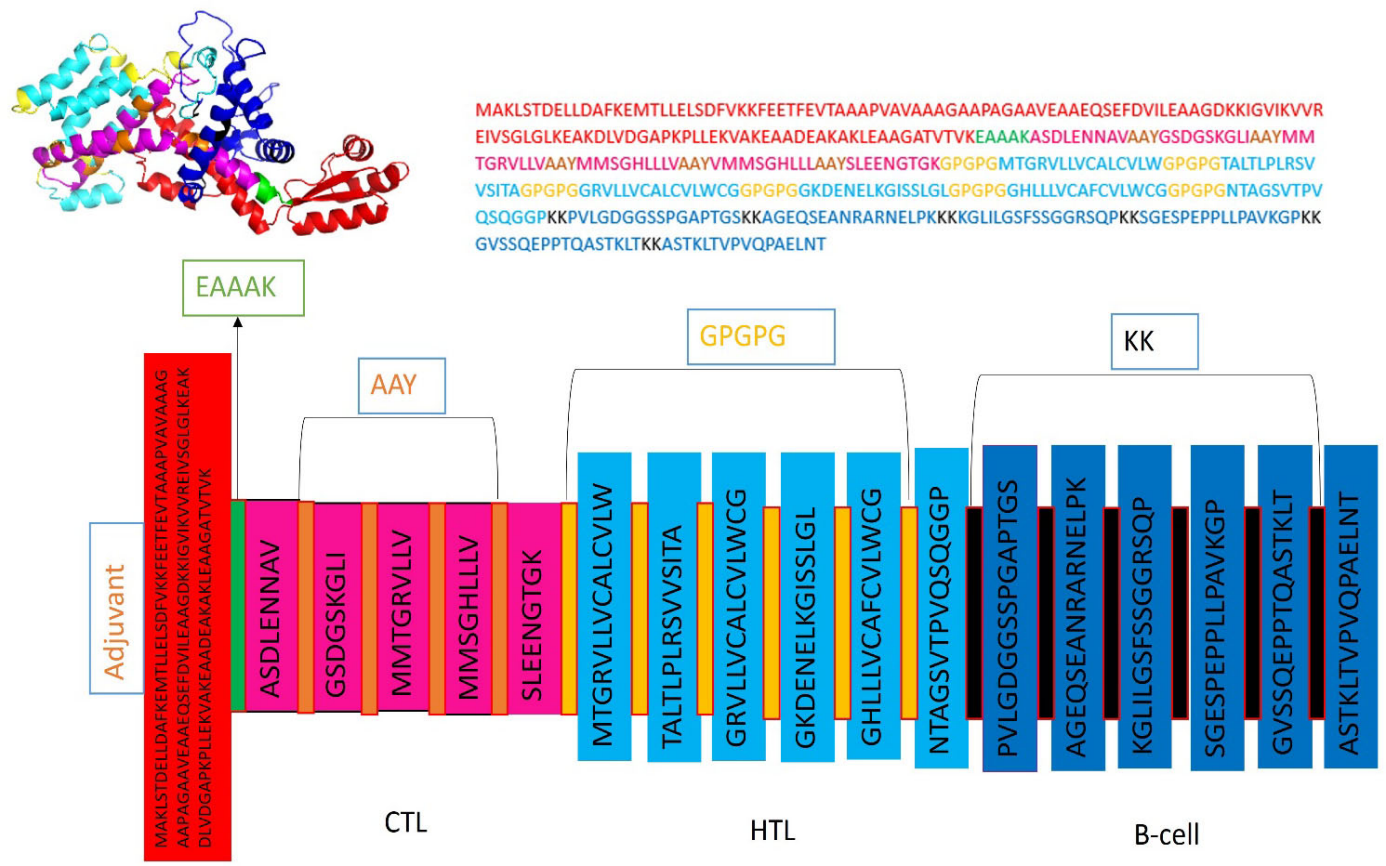
Graphical depiction of the placement of epitopes in the final vaccine design. Three-dimensional model of the MEVC, with each component represented by a distinct color.

Five CTL epitopes were chosen for each protein based on the highest COMB score, as shown in **Table 2**. A total of six non-allergenic HTL epitopes, with three selected for each protein, were chosen using percentile rank as indicated in **Table 2** followed by B-cell epitopes. The multi-epitope vaccination was supplemented with an adjuvant to enhance its immune response. The non-toxic *Mycobacterium tuberculosis* RGTB423 was linked as an adjuvant to the N-terminus of the vaccination sequence using an EAAAK linker. Junctional epitopes are not produced and the presentation of epitopes on receptors is enhanced due to their role as linkers. **Figure 2** illustrates that the ultimate vaccination sequence, encompassing the selected epitopes, had a length of 431 amino acids as shown in **Figure 3**.

### 3.4 Prognostication of underlying physiological characteristics

**Figure 4:**
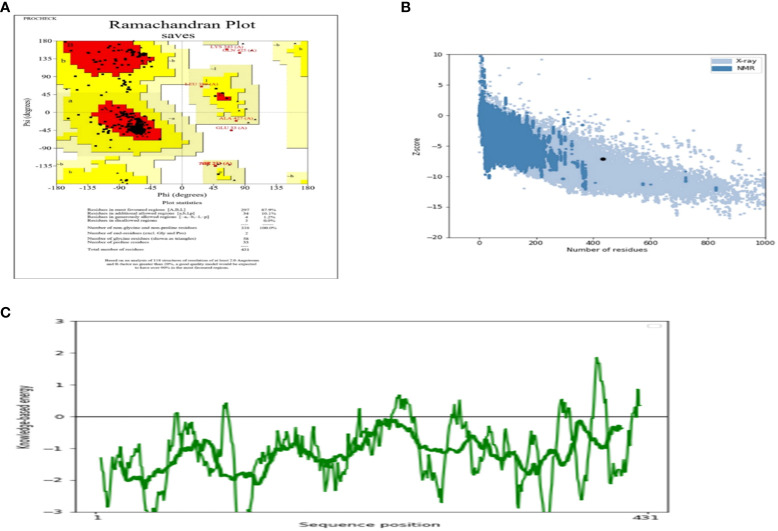
**(A)** Depicting the Ramachandra plot **(B)** presenting the z-score of the whole modelled vaccine construct **(C)** shows the sequence position with stable binding energies.

**Figure 5:**
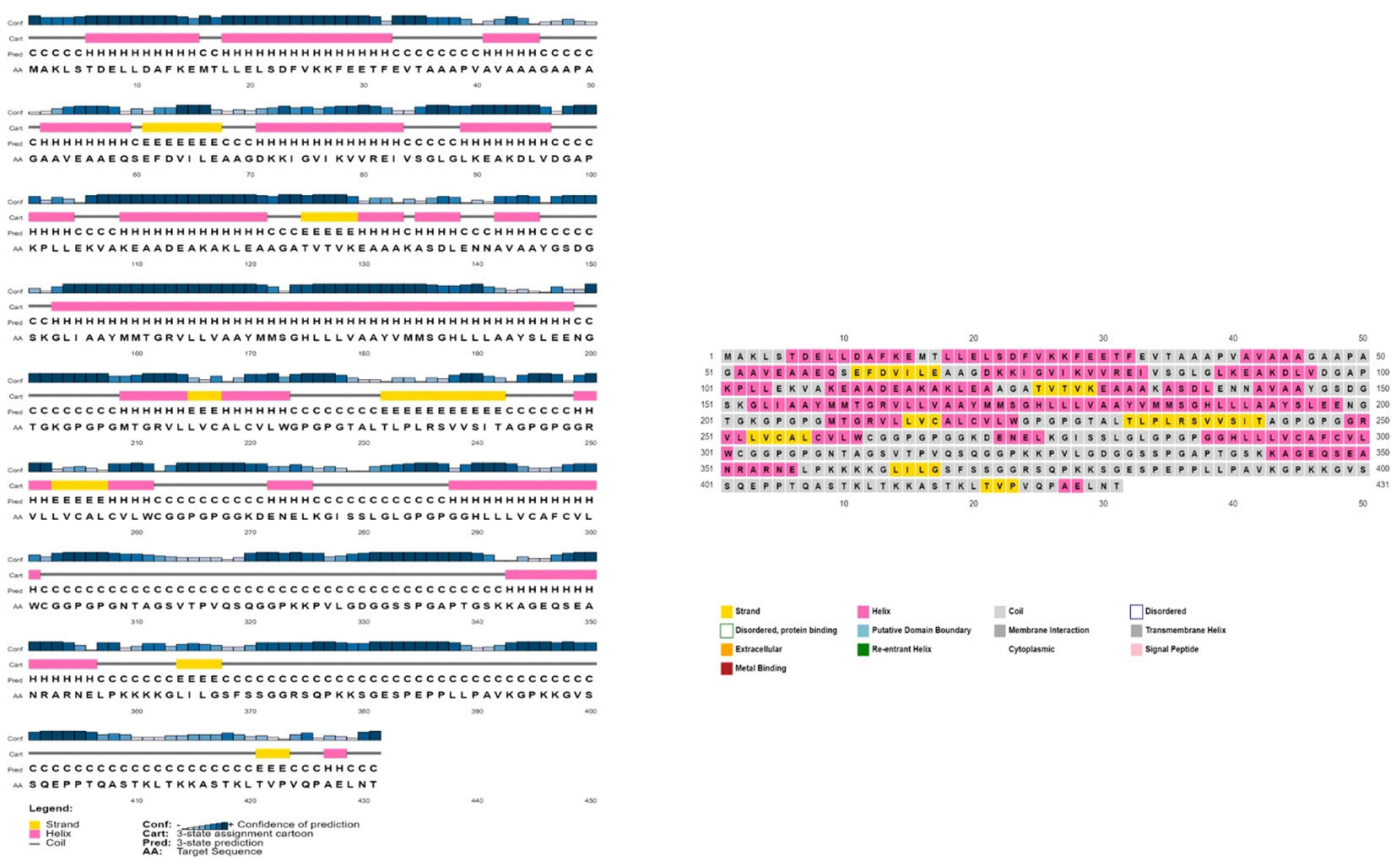
The provided visual representation illustrates the secondary structure obtained for the multi-epitope subunit vaccine construct, revealing the presence of alpha-helix beta strands, coils and other important domains.

The AlgPred server was utilized to verify the non-allergenic properties of the vaccination. The server reported an allergenicity score of -1.63, with a default threshold of -0.4. Simultaneously, the Vaxijen server evaluated the antigenic potential of MEV using a virus as the model organism. It assigned a score of 0.70 to MEV with a threshold of 0.4. The antigenic characteristics of the substance were confirmed. The physicochemical properties of the vaccination were determined using the ProtParam server. The determination of the molecular weight yielded a value of 43441.35 KDa, whereas the calculated theoretical isoelectric point (PI) was found to be 8.47. Moreover, the *in-vivo* half-life of *E. coli* exceeded 10 hours, suggesting a high level of stability. Furthermore, the stability of the vaccine was assessed using the instability index, yielding a value of 33.83, indicating stability (values below 40 are considered stable). In addition, it was found that MEVC has thermostability, as evidenced by its aliphatic index of 90.84. On a grand average, the vaccination had a hydropathy grade of 0.063. Based on the GRAVY values, it is possible that the protein possesses hydrophilic properties, indicating a tendency to engage in more favorable interactions with neighboring water molecules. The Procheck server was utilized to conduct a Ramachandran plot analysis, which revealed that 87.9% of the residues in the primary model were located inside the favored regions, 7.6% were situated within the additionally allowed regions, and 0.9% were found in the outlier disallowed regions as shown in **Figure 4A**. A stable residue with stable 3d structure has been inferred with z score in between -5 and -10 as shown in **Figure 4B** with **Figure 4C** showing the stable sequence position low binding energies for all the residues except few. The PSIPRED server was utilized to anticipate the secondary structure of the finalized vaccine build. According to the server’s data, the vaccine structure is believed to be composed of alpha-helix, coil forms, and beta strands, as depicted in **Figure 5**.

### 3.5 Molecular docking

**Figure 6:**
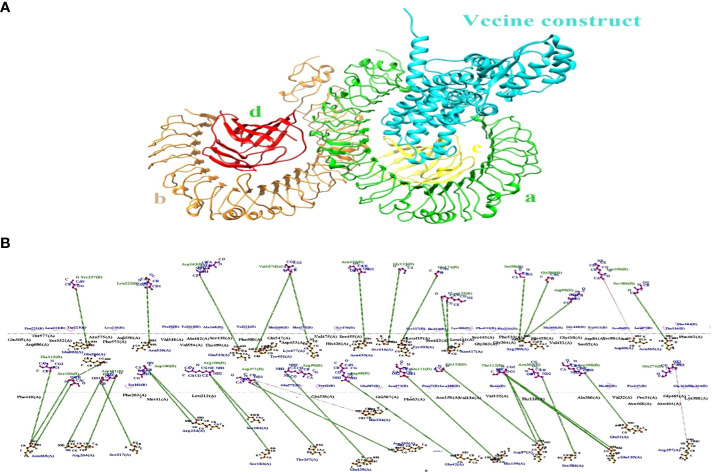
**(A)** The docking complex between human TLR4 and MEVC of *T. cruzi* is illustrated. **(B)** The MEVC residues interact with the TLR4 receptor protein through the interaction between chain A and chain B.

The binding effectiveness of MEVC against the human receptor protein TLR4 was observed using a molecular docking technique. To mitigate steric conflicts at the atomic level among residues, minimization was performed for both MEVC and the target protein prior to docking. The Cluspro 2.0 server (49) was utilized to conduct molecular docking analysis between the suggested vaccination and Toll-Like receptor 4 (TLR4) (Pdb Id: 3fxi).The 3-d docked complex structure shows the binding mode of the construct with the receptor target with most of the residues bind with chain A and C having binding energies of −258.7 kcal/mol as shown in **Figure 6A**. Schema diagrams depicting the bonds formed between vaccine construct residues and TLR4 were generated using the LigPlot software (50). Eleven clusters were generated by the server, and the number of members and minimum energy for each cluster were computed. Upon comparing all clusters, it was observed that Cluster 1 had the highest interaction energy, characterized by the most negative value of -1038.2 kcal/mol. The interconnections between the vaccine construct and receptor protein consist of the following residues: Glu603 (A)-Ser273 (B), Asn526 (A)-Leu222 (B), Glu523 (A)-Arg163 (B), Tyr451 (A)-Vall67 (B), Lys477(A)-Vall67 (B), Ser416 (A)-Gly23 (B), Tyr403 (A)-Lys125 (B) and so many others showing strong hydrogen bonds as shown in **Figure 6B**. Therefore, a molecular dynamic analysis was conducted to validate these linkages. The investigation followed a simulation time interval of 100ns and included stability checks and confirmational modifications.

### 3.6 Molecular dynamic simulations

**Figure 7:**
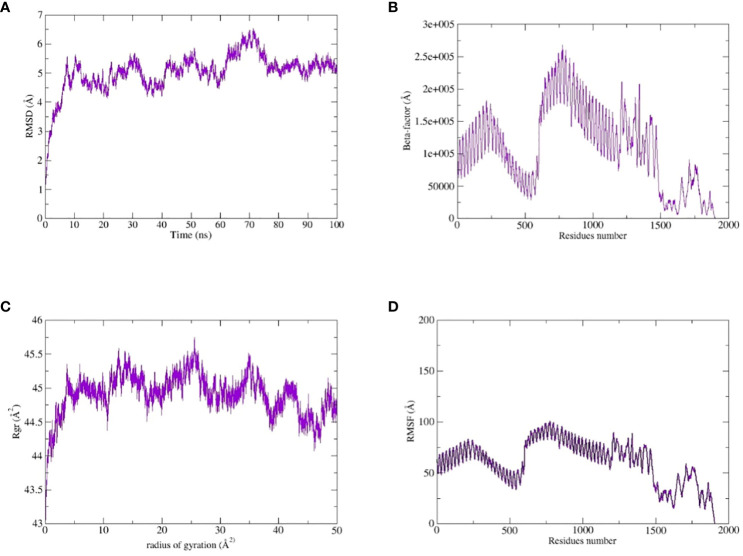
MD simulation study of the complexes of MEVC, TLR4: **(A)** the RMSD, **(B)** the Beta-factor, **(C)** the RoG plot and **(D)** RMSF.

**Figure 8:**
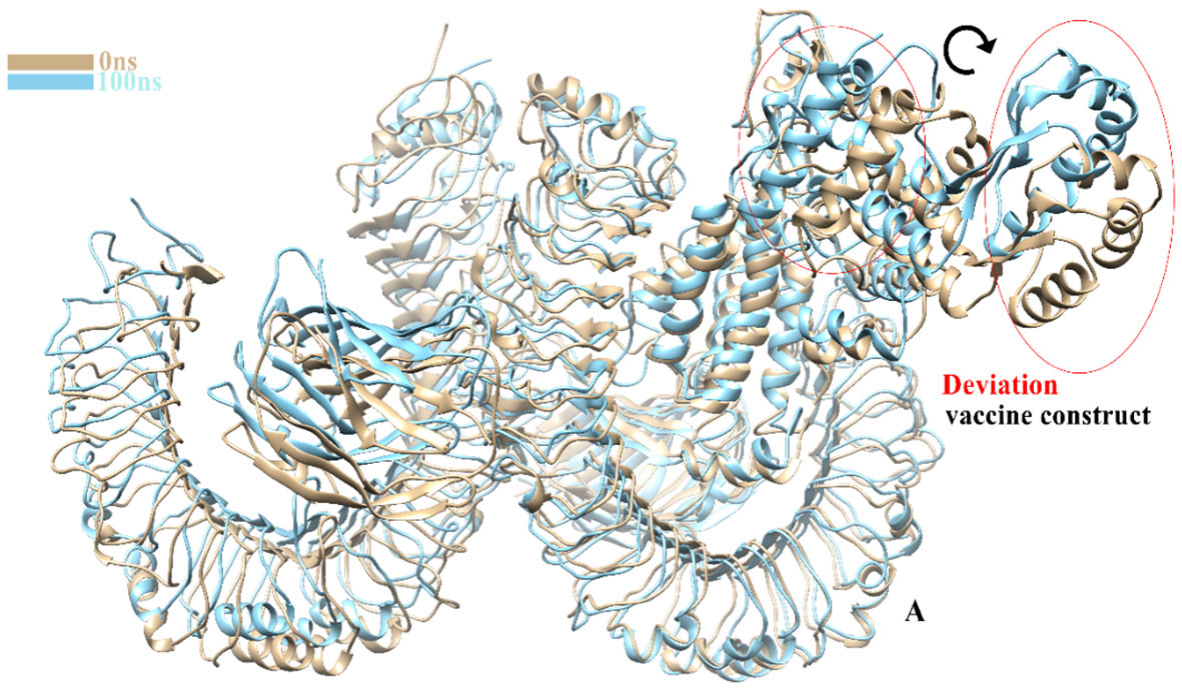
Clear image shows a slight change in the degree of MEVC at 0ns and 100ns.

The TLR-4-vaccine docked complex was subjected to molecular dynamics (MD) simulation in an explicit water environment for a duration of 100 nanoseconds. The stability of the simulation process was assessed by examining the deviation of backbone atoms of the complex using the root-mean-square-deviation (RMSD). The results demonstrated a consistent and steady behavior of the vaccinated TLR4 complex, as seen by the thin string-like pattern of RMSD. In the RMSD graph, there was some fluctuation observed at the start of the simulation time intervals and other different time intervals i.e. 8ns, 30ns, 50ns and 70ns. but no significant changes were observed in relation to the MD simulation after 70ns and the system remains stable. The complex has an average root mean square deviation (RMSD) of approximately 5.03 Å as shown in **Figure 7A**. The findings indicated local fluctuations in TLR4, whereas the binding residues of the protein (TLR4-vaccine) exhibited significant fluctuations. Furthermore, the B-factors were obtained by the analysis of simulated trajectories in order to investigate the regions of the complexes that exhibit high mobility (see **Figure 7B**). The beta factors for the MEV-TLR4 complexes exhibited average values of 1.09e+5 Å. The observed variation in the residues of TLR4 proteins indicates that the TLR4 was stabilized by the subsequent vaccines (**Figure 7B**). These findings indicate that the vaccination binding enhanced the stability of the TLR4 protein, making it one of the most effective vaccines for TLR4. On other hand radius of gyration (Rg), which was measured for the protein backbone, discusses how compact the protein is. The compactness of the protein during the simulation was determined by comparing the Rg, average mean value for Rg is 44.7 Å (**Figure 7C**). To assess the degree of variability in the residue within the TLR4-vaccine complex. The root-mean-square deviation (RMSF) was assessed to obtain further insights into the flexibility of each residue. The root mean square fluctuation (RMSF) and flexibility demonstrated that the MEV flexible zones are accountable for the greater flexibility observed in complex system with average RMSF of 49.50 Å as shown in **Figure 7D**. In addition, the simulation frames gained at the beginning of the simulation were superimposed onto the structures produced at the end frames as shown in **Figure 8**. The contacts between MEV and receptors remained consistently constant and actively engaged, as previously mentioned. This finding provides more clarification about the robust stability of these complexes across the whole 100 nanosecond simulation duration. Results for MD simulations at different intervals i.e. 0ns and 100ns also shows that a slight degree of diversion of vaccine construct occur at the surface of the target protein but remains static till the end of the time intervals as shown in **Figure 8**.

### 3.7 Immune simulation

**Figure 9:**
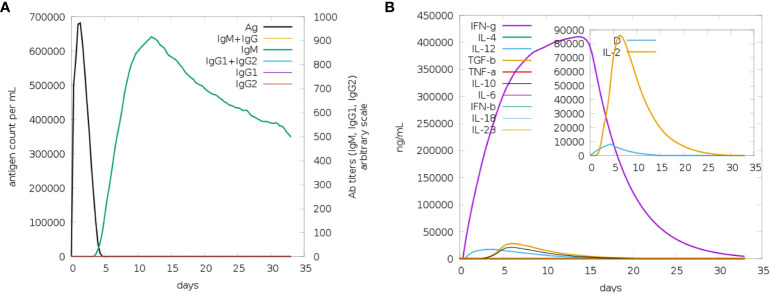
**(A)** The generation of immunoglobulins and B-cell isotypes exposure to MEV. **(B)** Additionally, the production of interleukins and cytokines with varying Simpson index in different states of MEV exposure.

It appears that both secondary and primary immune responses play a significant role in the pathogens and are likely to contribute to the overall immune response. **Figure 9** depicts the reaction of the *in-silico* host immune system to the antigen. The main reaction was characterized by elevated levels of IgM and IgG + IgG, followed by IgG1 and IgG1 + IgG2, and IgM **Figure 9A**, during both the primary and secondary stages, accompanied by antigen decrease. Additionally, a robust response of cytokines and interleukins has been reported. Likewise, a significant rise in IFN-g (>400,000 ng/ml) was seen for about 33 days (**Figure 9B**). Based on future experiences, it can be inferred that the immunological response and acceptance of MEV have been successful.

### 3.8 *Insilico* cloning

**Figure 10:**
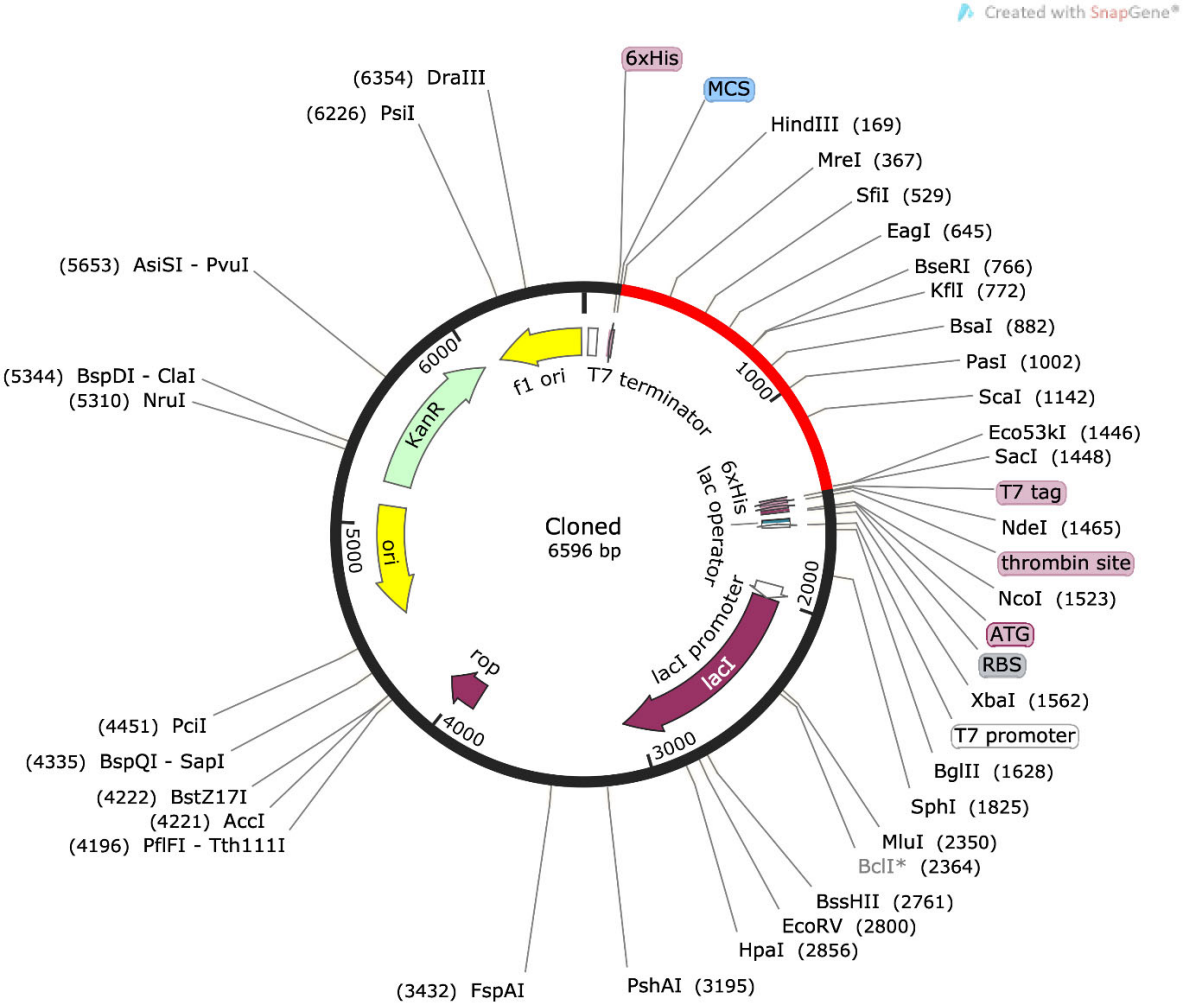
The vaccine design is cloned into pET-28a(+) is shown in red color.

For maximal expression in the *E. coli* expression system, *insilico* cloning of the disulfide-engineered construct was accomplished. To do this, the build sequence was first made better for the CAI-Value, from which the ideal value is 0.87 where is the construct GC content is 61.27% as shown in **Figure 10**.

### 3.9 PCA analysis

**Figure 11:**
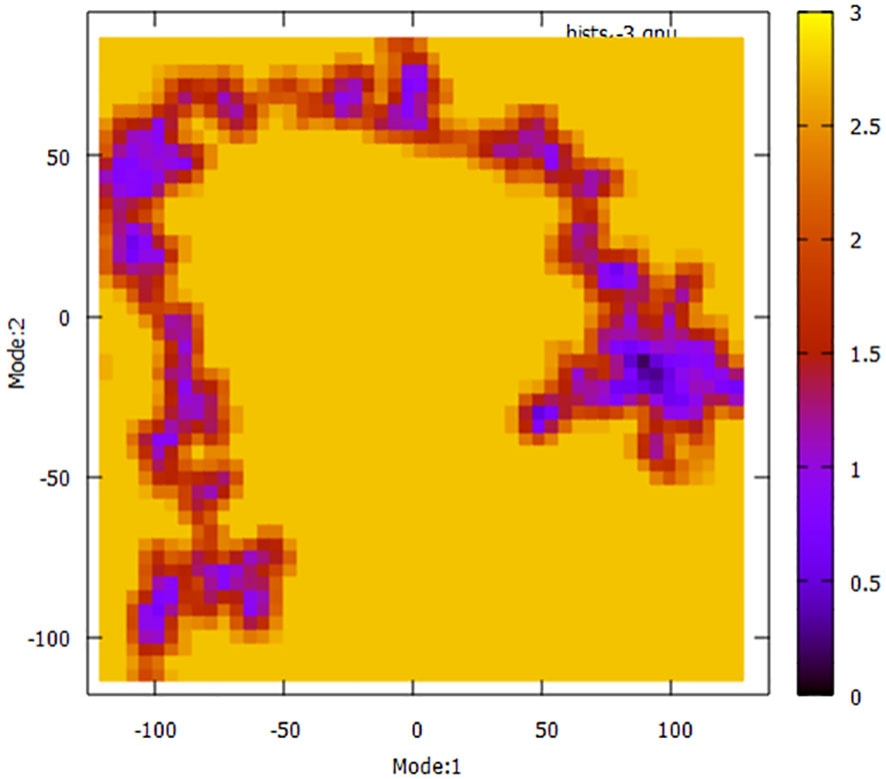
Principal Component Analysis (PCA) techniques, with the aim of demonstrating the influence of extended simulation duration based on energy landscape.

To have a more comprehensive understanding of the dynamic motion of the protein, it is crucial to conduct a thorough study of its key dynamics. Proteins typically display a certain degree of flexibility and rigidity, especially in relation to the residues situated near the binding pocket. To gain a comprehensive understanding of the protein’s overall motion observed during the simulation run, the dimension reduction technique known as essential dynamics (ED) was employed for the initial two principal components (PCs). The covariance matrix of eigenvectors was diagonalized to comprehensively elucidate the subspaces in which protein dynamics takes place. The experimental findings indicate that the protein has undergone extensive movement through Mode1 before attaining the equilibrated state in the case of PC2 (**Figure 11**). This phenomenon is also evident in the context of the free energy sector. The present research demonstrates that the protein has traversed many energy basins, as evidenced by the presence of favorable conformations (indicated by the hue red to yellow).

### 3.10 Estimation of MMGBSA binding energy

The MEV-TLR4 complex’s binding free energies were determined using the MMGBSA technique. It was discovered that this compound had a Gibbs free energy of -54.6337 kcal/mol. Notably, the non-polar solvation energy had a relatively moderate influence at −113.1521 kcal/mol, but the electrostatic and van der Waals energies contributed considerably, totaling −253.5751 kcal/mol. It’s interesting to see that polar solvation energy contributed less favorably to the total net energy as shown in **Table 3**.

**Table 3:** Binding free energies (kcal/mol) estimation of bounded MEV to the human TLR4.

Energy Components	Binding Free Energies (kcal/mol)
	MEV-TLR4
Δevdw	−253.5751
ΔEele	−1452.7422
Epolar.solv	1620.74
Enon-polar.solv	−113.1521
ΔGgas (GBSA)	−1810.2374
ΔGsol (GBSA)	1647.404
ΔGbind (GBSA)	−54.6337

## 4 Conclusions

To identify immunodominant epitopes capable of activating both cellular and humoral immune responses to combat the infection, the whole proteomes were examined. Several immunoinformatics methods were used to identify potential vaccination candidates. A stable physicochemical profile, high water solubility, antigenicity, and lack of allergenicity were demonstrated by the developed vaccine construct. For preventive and therapeutic vaccine formulations, the vaccine design includes immunogenic, presumably safe, and harmless epitopes. In order to trigger a timely response and enable the establishment of adaptive immunity against the pathogen, it has been demonstrated that the vaccine design binds to the innate immune TLR4 receptor with high affinity. This study employed various computational techniques to discover probable B- and T-cell epitopes in the *T. cruzi* core proteome. The vaccine candidate that has been developed has immunodominant characteristics. Computational investigation revealed that Chagas illness elicited a robust immunological response by binding to TLR4. Based on the results of our research, it is our contention that the initial step in developing a vaccine candidate targeting the causative agent of Chagas disease in humans should involve the formulation of the candidate vaccine. Furthermore, the epitopes identified in this study have the potential to be utilized in subsequent investigations, although further evidence is required to validate the immunogenicity and effectiveness of the anticipated vaccine. To establish the efficacy of our developed vaccine in providing comprehensive protection against Chagas disease, it is imperative to conduct a thorough *in vivo* investigation.

## Data availability statement

The original contributions presented in the study are included in the article/supplementary material. Further inquiries can be directed to the corresponding author.

## Author contributions

FA: Writing – original draft. AA: Writing – review & editing. HA: Writing – review & editing. KA: Writing – review & editing.
